# The Roles of Social Media Use and Friendship Quality in Adolescents’ Internalizing Problems and Well-being

**DOI:** 10.1007/s10902-022-00539-w

**Published:** 2022-06-06

**Authors:** Chantie Charissa Luijten, Daphne van de Bongardt, Anna Petra Nieboer

**Affiliations:** 1 Department of Socio-Medical Sciences, Erasmus School of Health Policy & Management, Erasmus University Rotterdam, P.O. Box 1738, 3000 DR Rotterdam, The Netherlands; 2 Department of Psychology, Education and Child Studies, Erasmus School of Social and Behavioural Sciences, Erasmus University Rotterdam, P.O. Box 1738, 3000 DR Rotterdam, The Netherlands

**Keywords:** Social media use, Friendship quality, Adolescence, Internalizing problems, Well-being, Gender

## Abstract

Adolescents spend increasing amounts of time using social media, but whether social media use has a beneficial or harmful role in internalizing problems and well-being during adolescence remains under debate. The present study explored associations of social media use and friendship quality with adolescents’ internalizing problems and well-being both concurrently and longitudinally, including the exploration of interactive effects between social media use and friendship quality and the examination of gender differences. Online questionnaire data collected in Spring 2018 and Spring 2019 from 1,298 Dutch adolescents aged 11–17 years (mean age 13.7 ± 1.1 years, 53.2% girls) were used. Path analyses showed that, cross-sectionally, girls (not boys) who used social media more frequently had more internalizing problems and lower well-being. Boys and girls with higher-quality friendships reported fewer concurrent internalizing problems and higher concurrent and longitudinal well-being; the association with internalizing problems was significantly stronger for girls as for boys. We found no significant interaction between social media use and friendship quality. Thus, the present study indicates that social media use and friendship quality have unique roles in adolescents’ internalizing problems and well-being. Furthermore, the findings support the importance of gender-specific approaches to decrease adolescents’ internalizing problems and enhance their well-being.

## Introduction

Since the early 2000s, adolescents have spent increasing amounts of time engaging with social media (Boyd & Ellison, 2007), defined broadly as media used for social interaction, or digital applications or tools that allow users to generate and share content and communicate with others (Carr & Hayes, 2015; Moreno & Kota, 2013). Today, most adolescents use social media (e.g., WhatsApp, Facebook, Instagram, and Snapchat); for instance, about 95% of Dutch adolescents (aged 12–18 years) report such use (Centraal Bureau voor de Statistiek, 2019) and, although use frequency varies widely among individuals, 31% use social media almost all the time throughout the day (Stevens et al., 2018). Thus, an increased understanding of the role of social media use in adolescents’ development, and specifically their mental health, is needed, as this factor is an important societal concern in research, practice, and public health policy (e.g., Clarke et al., 2015; Parkin et al., 2019).

In conceptualizing mental health, increasing attention is being paid to the integration of symptoms with strengths and the balance between risks and resources (Kobau et al., 2011; Peterson & Seligman, 2004). The dual-continuum model holds that mental health problems (e.g., internalizing problems, including depressive and anxiety symptoms) and well-being (i.e., life satisfaction, positive emotions, and good functioning in one’s individual endeavors and social life) (Diener, 2009; Gallagher et al., 2009) are related, yet distinct, continua, rather than opposite ends of a single continuum (Keyes, 2005). Concerns have been raised about the potential role of social media use in both adolescents’ internalizing problems and well-being, and recent research has indicated the relevance of distinguishing the two (Boer et al., 2020; Fardouly et al., 2018; Petropoulos Petalas et al., 2021).

As social media use is inherently social, and intertwined with the ways in which adolescents establish and maintain (offline) friendships, the consideration of friendships is important when examining the role of social media use in adolescents’ internalizing problems and well-being (Koo et al., 2015). Adolescents with higher-quality friendships report fewer internalizing problems (Schwartz-Mette et al., 2020) and higher well-being (Raboteg-Šarić & Šakić, 2014) than do those with lower-quality friendships. Moreover, the associations of social media use with adolescents’ internalizing problems and well-being may depend on adolescents’ friendships. Particularly friendship quality, characterized by positive (e.g., warmth) and negative (e.g., conflict) aspects (Furman & Buhrmester, 2009), has been found to affect (moderate) associations among adolescents’ social media use, internalizing problems, and well-being (Selfhout et al., 2009; Valkenburg & Peter, 2007).

Within the social media literature, four theoretical hypotheses are discussed regarding the interactive effects of social media use and friendship quality. The *rich-get-richer* hypothesis suggests that more-frequent social media use has the most-beneficial effects for adolescents with higher-quality friendships, as these media are ideal for the maintenance of these friendships and facilitate social network expansion for these adolescents, who may have better social skills that can be used to connect with new friends online (Kraut et al., 2002). The *poor-get-poorer* hypothesis suggests that more-frequent social media use has more-detrimental effects for adolescents with lower-quality friendships, who may have poorer social skills and be more likely to use social media to escape from real-life problems, which could have negative outcomes (Selfhout et al., 2009). The *social compensation* or *poor-get-richer* hypothesis suggests that adolescents with lower-quality friendships benefit from more-frequent social media use because fewer constraints that lead them to interact poorly in real-life face-to-face encounters with their friends are present in the online environment (Valkenburg & Peter, 2007). The *rich-get-poorer* hypothesis suggests that more-frequent social media use is harmful for adolescents with higher-quality friendships because it may reduce opportunities to maintain (offline) friendships or because these adolescents have less to gain from such use than do adolescents with lower-quality friendships (Lin et al., 2018).

In the present study, we first aimed to increase our understanding of the simultaneous associations of friendship quality and social media use with adolescents’ internalizing problems and well-being, both concurrently and longitudinally. In support of prior research, we hypothesized that higher-quality friendships would be related to fewer internalizing problems and higher well-being among adolescents (Raboteg-Šarić & Šakić, 2014; Schwartz-Mette et al., 2020). Despite the increasing body of research on associations of social media use with adolescents’ internalizing problems and well-being, systematic reviews reveal little agreement on whether effects of social media use on these factors are beneficial or harmful (e.g., Seabrook et al., 2016) and associations found are often small (e.g., Orben & Przybylski, 2019). Therefore, we expected that the role of social media use could be either negative (i.e., resulting in more internalizing problems and lower well-being) or positive (i.e., resulting in fewer internalizing problems and higher well-being).

In addition, we aimed to examine interactive effects of social media use and friendship quality based on the abovementioned theoretical hypotheses from the literature on social media use. Empirical results of the examination of these hypotheses, mostly of the rich-get-richer versus social compensation hypothesis, are mixed, and the identified interaction effects between social media use and friendship quality have been weak. For instance, a longitudinal study showed that social media use (measured as Twitter activity) was associated with fewer internalizing problems among adolescents with lower perceived friendship quality than among those with higher perceived friendship quality (Cole et al., 2019), thus supporting the social compensation hypothesis. Cross-sectional research has provided support for the rich-get-richer hypothesis, showing that the benefits of social media use are greater for adolescents who have good offline friendships than among those who do not (Khan et al., 2016). The present study contributed to the existing body of literature by exploring the applicability of all four hypotheses in characterizing the interaction between social media use and friendship quality in one study, while investigating concurrent and longitudinal associations with adolescents’ internalizing problems and well-being.

Furthermore, the impacts of social media use (Ivie et al., 2020; Orben, 2020; Sarmiento et al., 2018; Schønning et al., 2020) and friendship quality (Raboteg-Šarić & Šakić, 2014; Schwartz-Mette et al., 2020) on adolescents’ internalizing problems and well-being may differ by gender. Girls are typically socialized to value social relationships more than boys (You et al., 2018) and they appear to be more sensitive to social influences than boys (Cialdini & Trost, 1998; Rudolph & Conley, 2005). For instance, friendship quality has been found to be more important for the psychological well-being of girls compared to that of boys (Almquist et al., 2014). Girls are also argued to be more prone than boys to experience adverse effects of more-frequent social media use on their internalizing problems and well-being (Nesi & Prinstein, 2015). However, recent reviews of adolescents’ social media use show that such gender differences are understudied. As girls report more social media use, greater friendship quality, more internalizing problems, and lower well-being than do boys (Bartels et al., 2013; Kelly et al., 2018; Raboteg-Šarić & Šakić, 2014; Stevens et al., 2018), we hypothesized that associations of social media use and friendship quality with adolescents’ internalizing problems and well-being would be stronger for girls than for boys.

## Methods

### Participants

The present study was part of a larger two-wave longitudinal project on the socioecological predictors of the well-being of adolescents in the Netherlands (Luijten et al., 2019; 2021a; 2021b). With the schools’ provision of informed consent, adolescents from three secondary schools located in the areas of two large cities in the Netherlands (Amsterdam and Rotterdam) participated in the two study waves in spring 2018 (T1; grades 7–9) and spring 2019 (T2; grades 7–10). In each wave, adolescents and their parents were sent emails describing the study objectives and procedure, with an invitation to participate. The adolescents and their parents could decline study participation; 6.2% (*n* = 84) and 1.0% (*n* = 13), respectively, did so at T1, and 0% and 0.8% (*n* = 11), respectively, did so at T2. The medical ethics committee of Erasmus Medical Centre (Rotterdam) determined that the Medical Research Involving Human Subjects Act was not applicable to this study (protocol no. MEC-2018–055).

Overall, 1,304 adolescents (53.0% girls, mean age 13.8 ± 1.1 years) participated in the study (T1: *n* = 1,124, 53.1% girls, mean age 13.7 ± 1.1 years; T2: *n* = 1,055, 55.4% girls, mean age 14.6 ± 1.1 years). Most (*n* = 875, 82.9%) T2 participants had participated at T1. The sample analyzed in the present study comprised 1,298 adolescents (53.2% girls, mean age 13.7 ± 1.1 years), as 6 participants were excluded due to missing data on the study variables of interest. About three-quarters of the participants were enrolled in higher senior general/pre-university (73.3%) and about one quarter were enrolled in lower pre-vocational (26.7%) education. Based on birthplace information for the students and their parents, 57.0% of the participants had Western (Europe, the United States, Canada, Australia, and New Zealand) ethnocultural backgrounds and 43.0% had non-Western (Africa, the Middle East, Asia, and Latin and South America) backgrounds. Most of the participants were living with both parents (72.9%), followed by those living with one parent (15.0%), those having separated parents with co-parenting (6.7%), and those living in other situations (i.e., with one parent and his or her new partner [4.2%], with foster parents [1.0%], or on their own [0.1%]).

### Procedure

For both study waves, the lead researcher (the first author) and trained research assistants visited the participants’ classes, introducing the study, answering questions, and asking them to fill out online questionnaires. The researchers were present during questionnaire administration and ensured the participants’ privacy and data confidentiality. After filling out the questionnaire, the participants received small, non-financial rewards (e.g., candy), the lead researcher’s contact information in case of questions, and a list of websites providing information on the questionnaire topics. They were also entered into raffles for one gift (e.g., iPhone, PlayStation) per school and one gift card (€5–10, depending on grade) per class.

### Measures

#### Well-Being

The 14-item Mental Health Continuum–Short Form (MHC-SF) (Keyes, 2005) was used to assess adolescents’ emotional (three items), psychological (six items), and social (five items) well-being. Respondents were asked to report the frequency with which they had felt corresponding well-being aspects (e.g., happiness, responsibility management, and contributions to society) in the past month using a six-point scale ranging from 0 (never) to 5 (every day). Mean scores were calculated, with higher scores indicating higher levels of well-being. The MHC-SF has been validated for use with Dutch adolescents (Luijten et al., 2019) and showed good reliability in the current study (Cronbach’s *α* = 0.91 at T1, 0.92 at T2).

#### Internalizing Problems

The Revised Child Anxiety and Depression Scale-25 (RCADS-25) (Ebesutani et al., 2012) was used to assess the participants’ internalizing problems, based on anxiety (15 items; e.g., “I worry about things”) and depression (10 items; e.g., “Nothing is much fun anymore”) symptoms on a four-point scale ranging from 0 (never) to 3 (always). The reliability and validity of the two subscales have been confirmed, and total (summed) RCADS-25 scores have been shown to reliably reflect internalizing problems (Ebesutani et al., 2012). Higher total scores indicate greater symptom frequency. The RCADS-25 showed good reliability in the current study (Cronbach’s *α* = 0.91 at T1, 0.92 at T2).

#### Social Media Use

Social media use was measured with an item that is often used in scientific studies to measure general social media use: “How much time do you spend on social networking sites or apps like WhatsApp, Facebook, Instagram, and Snapchat?” (e.g., Bevelander et al., 2018; Marino et al., 2016, 2020). Responses were structured by a five-point scale (1 = never, 5 = always).

#### Friendship Quality

The three-item satisfaction (e.g., “How satisfied are you with the relationship with your close friends?”) and conflict (e.g., “How much do you and your close friends argue with each other?”) subscales of the Network of Relationships Inventory (Furman & Buhrmester, 2009) were used to assess the overall quality of adolescents’ close friendships. These subscales have often been applied to the examination of parental (e.g., Van de Bongardt et al., 2016; Zhang et al., 2018) and peer (e.g., Zhang et al., 2018) relationship quality. Item responses are structured by a six-point scale ranging from 1 (none) to 6 (the most). Conflict item scores were inverted so that higher mean scores reflected greater relationship quality (e.g., Zhang et al., 2018). In the current study, this six-item inventory showed good reliability (Cronbach’s *α* = 0.82 at T1, 0.81 at T2).

### Statistical Analyses

Using SPSS (version 27; IBM Corporation, Armonk, NY, USA), descriptive statistics were calculated for all variables. Mean T1 and T2 RCADS-25 and MHC-SF scores were compared using the paired-samples *t* test, and all mean scores were compared between boys and girls using the independent-samples *t* test. Bivariate Pearson correlations between the study variables were examined, with *r* values of 0.10–0.29, 0.30–0.49, and ≥ 0.50 considered to reflect weak, moderate, and strong correlation, respectively (Cohen, 1988). For all analyses, the alpha level was set to 5.0%.

The study hypotheses were tested with path models using structural equation modeling in R (version 4.0.3; R Core Team, 2020) with the *lavaan* package (Rosseel, 2012). To handle missing data (i.e., 13.9–19.1% of all total scores, attributable primarily to participant absences), the full information maximum likelihood (FIML) method (Enders & Bandalos, 2001) was used. This method allows for the use of all available data and provides more accurate results than listwise deletion (e.g., Enders & Bandalos, 2001). To account for non-normal data distributions, robust maximum likelihood estimation was performed with the calculation of robust standard errors and adjusted chi-squared values (Sass et al., 2014; Yuan & Bentler, 2000). We assessed model fit using the *χ*^*2*^ statistic, comparative fit index (CFI), root mean square error of approximation (RMSEA), and standardized root mean square residual (SRMR). Good model fit was indicated by CFI > 0.90, RMSEA < 0.08, and SRMR ≤ 0.08 (Bentler & Bonnet, 1980; Hu & Bentler, 1999).

We tested cross-sectional (T1) and longitudinal (T1–T2) models of associations with adolescents’ internalizing problems (model A) and well-being (model B). All potential predictors were measured at T1. Initial levels of internalizing problems and well-being at baseline were included as covariates in the longitudinal models. Because of the documented relevance of gender, age, ethnocultural background, and education level to the concepts investigated (Kriesi et al., 2012; Orben, 2020; Salmela-Aro & Tynkkynen, 2010; Vacek et al., 2010; Yucel & Yuan, 2016), these sociodemographic variables were included as covariates in all models. Concurrent correlations between the covariates and the predictors were allowed, except for the correlation between covariates age and ethno-cultural background.

We tested two forms of models A and B: model 1 was used to examine the main effects of social media use and friendship quality, and model 2 additionally included the interaction term of social media use × friendship quality. With both models, we also tested two-way interactions of gender with social media use and friendship quality (i.e., gender × social media use and gender × friendship quality). The three-way interaction term of gender × social media use × friendship quality was also added to model 2. All continuous independent variables were centered to minimize multicollinearity. For significant interaction terms, we conducted stratified follow-up analyses to gain further insight.

## Results

### Sample Characteristics

Most adolescents in the sample used social media; according to self-reports, 1.4% of adolescents never, 7.8% almost never, 27.8% sometimes, 53.6% often, and 9.3% always used social media at T1. On average, the adolescents in our sample scored 3.6 on this 5-point scale, suggesting an average use of social networking sites or social medial apps between ‘sometimes’ and ‘often’, leaning slightly more toward ‘often’. Girls reported more frequent social media use, higher friendship quality, and more internalizing problems than did boys, whereas boys reported higher well-being than did girls (Table 1). Between T1 and T2, boys’ and girls’ internalizing problems increased significantly (both *p* < 0.05) and girls’ (but not boys’) well-being decreased significantly (*t*[483] = 2.74, *p* = 0.006; Table 1).

**Table 1:** Mean social media use, friendship quality, well-being, and internalizing problems scores

	Mean (*SD*)		
	Total sample	Girls	Boys
Social media use T1	3.62 (0.82)	3.80^a^ (0.72)	3.40 (0.87)
Friendship quality T1	4.97 (0.67)	5.03^a^ (0.68)	4.89 (0.65)
Internalizing problems T1	11.45 (9.16)	13.70^a^ (10.00)	8.43 (6.93)
Internalizing problems T2	13.00 (10.54)	15.75^a^ (11.11)	9.76 (8.48)
Well-being T1	3.37 (0.97)	3.26 (0.96)	3.52^b^ (0.99)
Well-being T2	3.31 (0.98)	3.14 (0.96)	3.53^b^ (0.95)

Independent-sample *t* tests showed that all means at T1 and T2 differed significantly between girls and boys (*p* < 0.001). Paired-sample *t* tests showed that internalizing problems increased significantly (*t*[871] = − 5.86, *p* < 0.001) and well-being decreased significantly (*t*[869] = 1.97, *p* = 0.049) between T1 and T2 in the total sample.

*SD,* standard deviation; T1, spring 2018; T2, spring 2019

^a^Larger mean scores for girls

^b^Larger mean scores for boys

Significant correlations among social media use, friendship quality, and internalizing problems were found at T1 and T2 (Table 2). Correlations with social media use were positive, whereas those between friendship quality and internalizing problems were negative. At T1 and T2, friendship quality, but not social media use, correlated positively with well-being (Table 2). Most correlations were weak to moderate.

**Table 2:** Pearson correlations between variables of interest at T1 and T2

	1	2	3	4	5
1. Social media use T1	–				
2. Friendship quality T1	0.11***	–			
3. Internalizing problems T1	0.12***	−0.24***	–		
4. Well-being T1	−0.03	0.30***	−0.50***	–	
5. Internalizing problems T2	0.09**	−0.15***	0.69***	−0.36***	–
6. Well-being T2	−0.00	0.22***	−0.44***	0.61***	−0.57***

**p < 0.01

***p < 0.001

T1, spring 2018; T2, spring 2019

### Model A: Internalizing Problems

The cross-sectional version of Model A fitted the data well (*χ*^*2*^(1) = 3.73, *p* = 0.053, CFI = 0.99, RMSEA = 0.05, SRMR = 0.01). Cross-sectionally, social media use, friendship quality, and gender were related significantly to the participants’ internalizing problems, whereas covariates age, ethnocultural background, and educational level were not (Table 3). More internalizing problems were reported by girls, more-frequent social media users, and adolescents with lower-quality friendships. The association with friendship quality was significantly stronger than that with social media use (*p* < 0.001), and the interaction between these variables was not significant (B = −0.06, SE = 0.43, β = −0.00, *p* = 0.895). Accordingly, model fit did not change after adding the interaction effect.

**Table 3:** Cross-sectional and longitudinal main effects of social media use on adolescents’ internalizing problems and well-being

	Model A						Model B					
	Internalizing problems T1			Internalizing problems T2			Well-being T1			Well-being T2		
	B	SE	β	B	SE	β	B	SE	β	B	SE	β
Intercept	13.54	0.75	1.49***	13.47	0.69	1.31***	3.30	0.08	3.37***	3.33	0.07	3.46***
Gender	−5.42	0.50	−0.30***	−1.83	0.53	−0.09**	0.30	0.06	0.16***	0.25	0.05	0.13***
Age	0.04	0.23	0.01	−0.57	0.23	−0.06*	−0.02	0.03	−0.03	0.06	0.02	0.07*
Ethno-cultural background	−0.28	0.54	−0.02	0.14	0.54	0.01	0.06	0.06	0.03	−0.03	0.05	−0.02
Educational level	0.47	0.64	0.02	0.40	0.61	0.02	−0.11	0.07	−0.05	−0.13	0.07	−0.06
Internalizing problems T1				0.77	0.04	0.67***						
Well-being T1										0.58	0.03	0.58***
Social media use	0.84	0.31	0.08**	−0.18	0.33	−0.01	−0.03	0.04	−0.03	0.05	0.04	0.04
Friendship quality	−3.76	0.43	−0.28***	0.01	0.57	0.00	0.47	0.04	0.32***	0.10	0.05	0.07*

*p < 0.05

**p < 0.01

***p < 0.001

T1, spring 2018; T2, spring 2019; *SE,* standard error

The longitudinal version of Model A also showed good model fit (*χ*^2^(1) = 3.71, *p* = 0.054, CFI = 1.00, RMSEA = 0.05, SRMR = 0.01). Age, gender, and internalizing problems at T1 were significant covariates in the longitudinal model; ethnocultural background and education level were not (Table 3). Girls, younger adolescents, and adolescents with more internalizing problems at T1 reported more internalizing problems at T2 than did boys, older adolescents, and those with fewer internalizing problems at T1, respectively. Although social media use and friendship quality were associated significantly with adolescents’ internalizing problems concurrently, they were not related significantly to adolescents’ internalizing problems one year later. After adding the interaction between social media use and friendship quality model fit did not change and the interaction effect was not significant (B = 0.20, SE = 0.54, β = 0.01, *p* = 0.718).

### Model B: Well-Being

The cross-sectional version of Model B fitted the data well (*χ*^2^(1) = 3.71, *p* = 0.054, CFI = 0.99, RMSEA = 0.05, SRMR = 0.01). In the cross-sectional model, gender and friendship quality were related significantly to adolescents’ well-being, whereas age, ethnocultural background, educational level, and social media use were not (Table 3). Boys and adolescents with higher-quality friendships reported significantly higher well-being levels. Adding the interaction between social media use and friendship quality did not change model fit, as the interaction effect was not significant (B = − 0.06, SE = 0.05, β = − 0.03, *p* = 0.245).

The longitudinal version of Model B also revealed good model fit (*χ*^*2*^(1) = 3.70, *p* = 0.055, CFI = 1.00, RMSEA = 0.05, SRMR = 0.01). In the longitudinal model, gender, age, and well-being at T1 were significant covariates; ethnocultural background and education level were not (Table 3). Thus, boys, older adolescents, and participants with higher well-being at T1 reported higher well-being at T2 than did girls, younger adolescents, adolescents with lower well-being at T1, respectively. In line with the cross-sectional model, adolescents with higher-quality friendships reported significantly higher levels of well-being one year later, and social media use was not related significantly to adolescents’ well-being over time. The interaction between social media use and friendship quality was not significant (B = −0.07, SE = 0.05, β = −0.04, *p* = 0.121). Correspondingly, the model fit did not change.

### Gender Differences

**Fig. 1:**
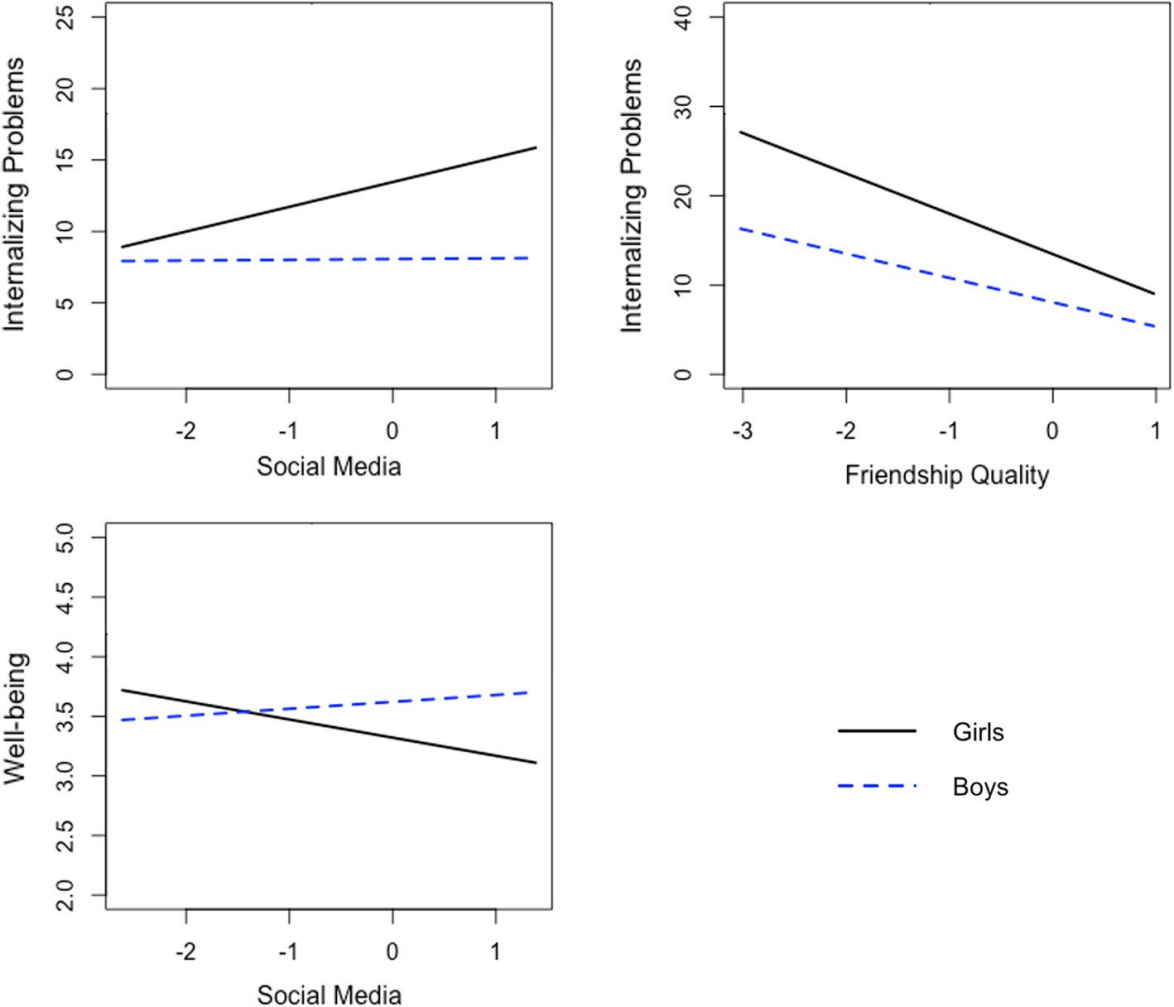
Significant two-way interaction effects of gender and social media use or friendship quality on adolescents’ internalizing problems and well-being

Cross-sectionally, we found significant interaction effects of gender with social media use (B = −1.68, SE = 0.62, β = −0.11, *p* = 0.007) and friendship quality (B = 1.82, SE = 0.83, β = 0.09, *p* = 0.028) for adolescents’ internalizing problems. Girls (but not boys) who used social media more frequently reported significantly more internalizing problems (B = 1.64, SE = 0.54, β = 0.12, *p* = 0.002; Fig. 1). Boys and girls with higher-quality friendships reported significantly fewer internalizing problems; this association was significantly stronger for girls (B = −4.49, SE = 0.66, β = −0.30, *p* < 0.001) than for boys (B = −2.73, SE = 0.49, β = −0.26, *p* < 0.001; Fig. 1). When including these significant gender interaction effects to the cross-sectional version of Model A, it showed satisfactory to good model fit (*χ*^*2*^(2) = 21.69, *p* < 0.001, CFI = 0.99, RMSEA = 0.09, SRMR = 0.04).

We also found a significant interaction effect between gender and social media use for well-being (B = 0.21, SE = 0.07, β = 0.13, *p* = 0.003), with girls (but not boys) who used social media more frequently reporting significantly lower well-being (B = −0.14, SE = 0.05, β = −0.10, *p* = 0.007; Fig. 1). The interaction effect between gender and friendship quality was not significant regarding adolescents’ concurrent well-being (*p* > 0.05). The cross-sectional version of Model B including these two gender interaction terms also showed satisfactory to good model fit (*χ*^2^(2) = 21.58, *p* < 0.001, CFI = 0.99, RMSEA = 0.09, SRMR = 0.04).

The three-way interaction of gender × social media use × friendship quality was not significant for adolescents’ concurrent internalizing problems or well-being. Accordingly, adding the three-way interaction effects revealed poor model fits for internalizing problems (*χ*^*2*^(5) = 487.98, *p* < 0.001, CFI = 0.76, RMSEA = 0.27, SRMR = 0.11) and well-being (*χ*^*2*^(5) = 488.85, *p* < 0.001, CFI = 0.75, RMSEA = 0.27, SRMR = 0.11). Longitudinal analysis revealed no significant two- or three-way interaction effect involving gender for internalizing problems or well-being.

## Discussion

As adolescents spend increasing amounts of time using social media, increasing our understanding of the effects thereof on adolescents’ internalizing problems and well-being is important (e.g., Clarke et al., 2015; Parkin et al., 2019). The present study explored associations of social media use and friendship quality with adolescents’ internalizing problems and well-being concurrently and longitudinally, with testing for gender differences and interaction effects. We found that adolescent girls (but not boys) who used social media more frequently reported more concurrent internalizing problems and lower concurrent well-being, and that adolescents with higher-quality friendships reported fewer concurrent internalizing problems (this association was about twice as strong for girls than for boys) and higher concurrent and longitudinal well-being. These findings emphasize the need to employ gender-specific approaches to identify and decrease adolescents’ internalizing problems and enhance their well-being, and the value of the dual-continuum perspective (i.e., separate examination of internalizing problems and well-being) (Keyes, 2005) for research of this nature.

Our findings that girls use social media more frequently and have more internalizing problems and lower well-being than do boys confirm previous findings (Bartels et al., 2013; Kelly et al., 2018; Stevens et al., 2018). In addition to these mean differences between boys and girls, researchers have argued that girls are more prone than boys to experience adverse effects of more-frequent social media use on their internalizing problems and well-being, for instance because they have greater tendencies to ruminate about social media content and to compare themselves (e.g., their body images) with others appearing online (Nesi & Prinstein, 2015; Santarossa & Woodruff, 2017). Other reasons might be that, compared to boys, social media activities of girls may result in unwanted situations more often, including experiences of sexual harassment (e.g., sexual comments or jokes; showing sexual pictures; or spreading sexual rumors; Baumgartner et al., 2010; De Graaf et al., 2017; Petersen & Hyde, 2013). Thus, although scholars have raised concerns about the negative effects of social media use among all adolescents (Primack & Escobar-Viera, 2017; Underwood & Ehrenreich, 2017), our findings build on prior research and indicate that more-frequent social media use poses concurrent risks for girls’ but not for boys’ internalizing problems and well-being.

We found no longitudinal effect of social media use on adolescents’ internalizing problems or well-being, which aligns with some recent studies (Beeres et al., 2020; Boer et al., 2020; Coyne et al., 2020) but challenges others (e.g., Kelly et al., 2018). In general, this finding demonstrates that our cross-sectional result that social media use poses risks for girls’ internalizing problems and well-being does not provide information about the temporal sequence of involved processes or underlying mechanisms (Orben & Przybylski, 2019). Hence, potential explanations for our findings remain speculative. Social media use may have only short-term effects on adolescents’ internalizing problems and well-being, which were not captured fully by assessment at a longer-term (1-year) interval in this study (Keijsers & Van Roekel, 2018). Another explanation might be that social media use can have negative as well as positive effects on adolescents’ internalizing problems and well-being over time, which then even each other out. The positive psychological approach emphasizes the theoretical importance of considering the benefits of social media use, in addition to the disadvantages, to fully understand its effects on adolescents (De Leeuw & Buijzen, 2016). Recent empirical research indeed showed that social media use has positive as well as negative effects on adolescents’ internalizing problems and well-being (Schønning et al., 2020; Seabrook et al., 2016; Wen et al., 2016). Additional longitudinal research on associations of adolescents’ social media use with their internalizing problems and well-being is needed and recommended to use more and shorter-time intervals and further explore the unique positive and negative effects of social media use.

Contrary to the theoretical hypotheses that we examined, which were based on theoretical and empirical research (e.g., Abbas & Mesch, 2018; Khan et al., 2016; Selfhout et al., 2009; Valkenburg & Peter, 2007), we found no significant interactions between social media use and friendship quality in relation to adolescents’ internalizing problems or well-being concurrently or longitudinally. Thus, in the present study, adolescents’ social media use and friendship quality did not mutually reinforce benefits (*rich-get-richer* hypothesis) or increase risks (*poor-get-poorer* hypothesis), nor did one compensate for (*poor-get-richer* hypothesis) or deteriorate (*rich-get-poorer* hypothesis) the other. Rather, we found that social media use and friendship quality play unique roles in adolescents’ internalizing problems and well-being. Moreover, we found a greater strength of associations of friendship quality than social media use with adolescents’ internalizing problems and well-being. Thus, building on previous theoretical and empirical findings (Gaertner et al., 2010; Raboteg-Šarić & Šakić, 2014; Schwartz-Mette et al., 2020), the present study indicates that high-quality (offline) friendships remain crucial to adolescents’ concurrent internalizing problems and their concurrent and over-time well-being, despite increasing social media use. Indeed, the associations with girls’ social media use in the current study were negative and significant but small in line with prior research (Orben & Przybylski, 2019). Furthermore, although we found no gender differences regarding the association between friendship quality and well-being, the concurrent association with internalizing problems was about twice as strong for girls than for boys. The fact that girls tend to co-ruminate (i.e., disclose and extensively discuss emotional problems in dyadic relationships) with friends more often than do boys (Smith, 2015), may affect the strength and over-time stability of the association between friendship quality and internalizing problems. Overall, these findings contribute to the existing body of literature indicating that adolescents’ social media use and their (offline) friendship quality play unique roles in boys’ and girls’ internalizing problems and well-being.

The strengths of the current study include the participation of a large, culturally diverse sample of adolescents, the separate consideration of internalizing problems and well-being, the performance of cross-sectional and longitudinal assessments, and the simultaneous examination of social media use and friendship quality while testing for gender differences. However, some limitations of the present study need to be considered. First, as this research was based on adolescents’ retrospective self-reports, our findings may be subject to response bias (Krumpal, 2013). Second, we used a single item to measure adolescents’ time spent using social media. However, evidence indicates that single-item measures have reliability and predictive validity comparable to those of multiple-item scales (Bergkvist & Rossiter, 2007; Wanous & Hudy, 2001), and the construct of social media use appears to be sufficiently homogeneous and clear to adolescents for adequate operationalization with a single item, as in previous research (e.g., Bevelander et al., 2018; Marino et al., 2016, 2020). As recent research suggests that not only the frequency of social media use, but also the problematic use of these media, affects adolescents’ internalizing problems and well-being (Boer et al., 2020), we recommend additional longitudinal and multi-method research conducted with broader measures of social media use, including the extent to which it is problematic. Third, without replication, the results of this study cannot be generalized beyond our non-clinical school-based sample of Dutch adolescents. Adolescents in the Netherlands have the highest ranked well-being globally, and better friendships than do adolescents in other Western countries (Bradshaw et al., 2013). Studies conducted with other (sub-)clinical and non-clinical adolescent samples may differ. For instance, compared with their non-clinical peers, adolescents with (sub-)clinical mental health problems (e.g., depression) use social media more frequently (Sampasa-Kanyinga & Lewis, 2015; Uçar et al., 2020), perhaps because they have an unmet need for support or are concerned about what their friends will think. Thus, we recommend further research to determine whether the effects of social media use on adolescents’ internalizing problems and well-being differs according to clinical status.

Notwithstanding these limitations, our findings are highly relevant for parents, teachers, and pediatric and mental health professionals, who must be aware of and address the risks that more-frequent social media use incurs for adolescent girls. We also recommend that mental health professionals treating adolescents prioritize the maintenance of these patients’ existing friendships and encouragement that they form new (offline) friendships. This approach aligns well with the positive psychological approach (De Leeuw & Buijzen, 2016; Kobau et al., 2011), which proposes the identification and promotion of adolescents’ strengths and resources to improve their well-being, even in the presence of risk factors (e.g., more-frequent social media use for girls). Moreover, these findings highlight the urgency of the need for policymakers to consider the effects of adolescent physical distancing during the current COVID-19 pandemic (Rogers et al., 2021), as online friendships do not replace (the benefits of) high-quality offline friendships.

## Data Availability

The data are available upon (reasonable) request.
